# Supplementary material for: Multiple lines of evidence for a hypervelocity impact origin for the Silverpit Crater
Source: Nat Commun. 2025 Sep 20;16:8312. doi: 10.1038/s41467-025-63985-z (PMC12450236; doi:10.1038/s41467-025-63985-z)
Supplement: Supplementary file 1 — Supplementary Information [file 41467_2025_63985_MOESM1_ESM.pdf]

Supplementary Information for:

Multiple lines of evidence for a hypervelocity impact origin for the Silverpit Crater

Authors: Uisdean Nicholson[1]*, Iain de Jonge-Anderson[1], Alex Gillespie[2], James Frankel[2], Thomas Kenkmann[3], Tom Dunkley Jones[4], Gareth S. Collins[5], Veronica Bray[6], Sean Gulick[7,8], Ronnie Parr[9], Falko Langenhorst[10]

Table S1: Processing workflow for NEP 3D seismic data.

**Acquisition (PGS)**	
Vessel	Ramform Hyperion
Survey type	Towed streamer
Source	4x 400 cu.in. separated by 62.5. Inset 130m into streamer array. 6.25m shot interval (sequenced source 1, 2, 3, 4)
Streamer(s)	9x 3km streamers. 50m separation. 2ms sample rate, 4000ms record length
Sail lines	NW-SE. Nominal fold = 80
**Processing (Shearwater)**	
Processing	Proprietary Pre-Stack Depth Migration (PSDM) sequence on 12.5m x 12.5m bin grid

Table S2: Numerical model parameters for reconnaissance impact simulation

**Symbol**	**Definition**	**Value**		
L	Impactor diameter (m)	160		
v_i	Impact velocity (km/s)	15		
T_{dec}	Decay time of acoustic vibrations (s)	4.8		
$\upsilon_{\lim}$	Kinematic viscosity of acoustically fluidized region (m^2/s)	6,000		
		Impactor	Chalk	Clay/mudstone
ρ	Reference density (kg/m^3)	3300	2600	2650
Y_{i0}	Cohesion* (intact; MPa)	-	10	0.1
Y_m	Maximum shear strength (MPa)	3,500	100	1
μ_i	Coefficient of friction (intact)	-	1.2	1.0
Y_{d0}	Cohesion* (damaged; kPa)	10	100	1
μ_d	Coefficient of friction (damaged)	0.6	0.4	0.4
Y_T	Tensile strength (MPa)	0.01	10	0.1
p_{bd}	Brittle-ductile transition pressure (MPa)	-	185	1.73
p_{bp}	Brittle-plastic transition pressure (MPa)	-	220	2.07

*Yield strength at zero pressure

Figure S1: Uninterpreted and interpreted version of Figure 2. Panels a and b correspond to Figure 2a and 2b respectively, and seismic line locations are shown in Figure 1.

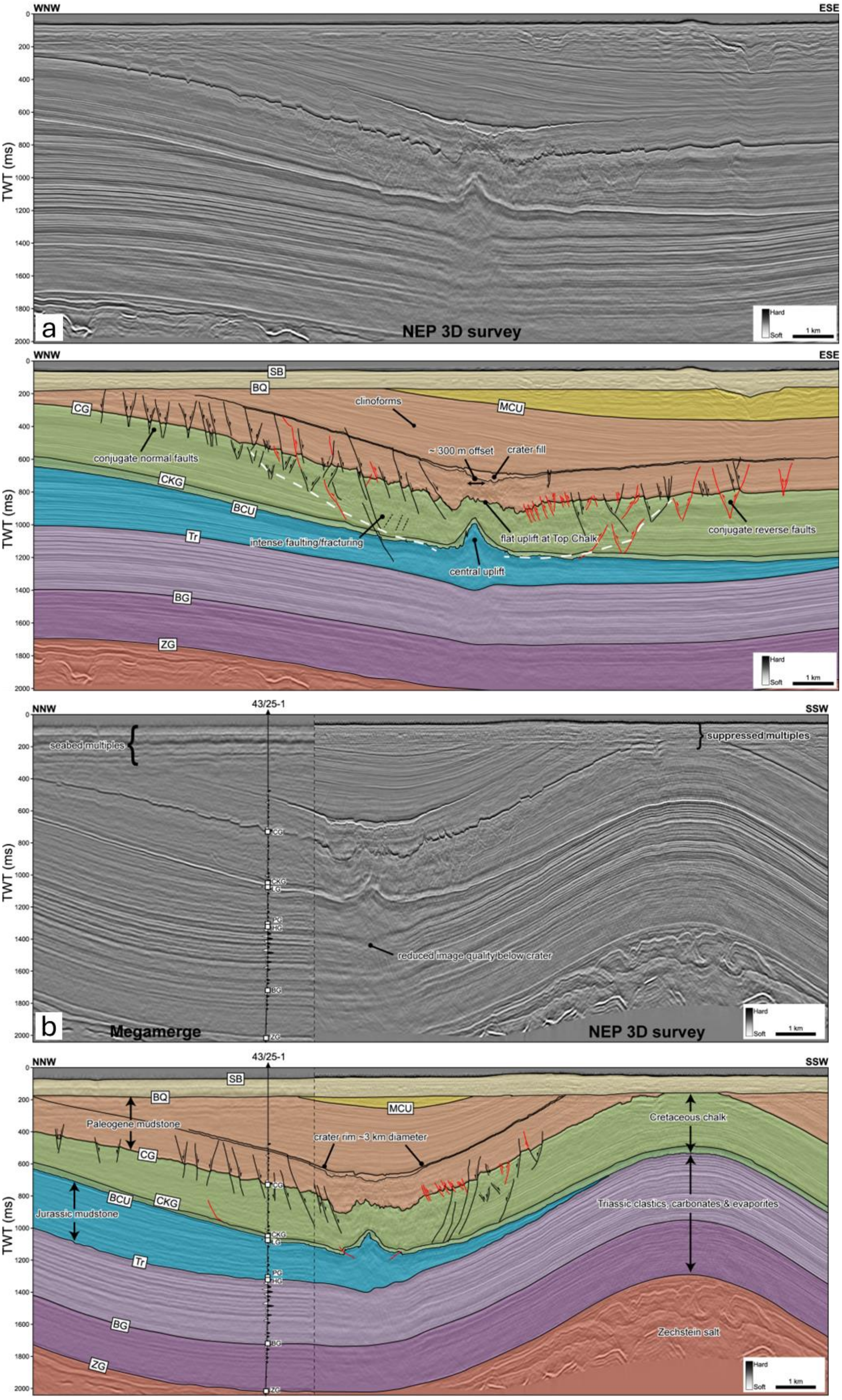

Figure S2: Plot of measured depth versus two-way-time for well 43/25-1 illustrating the three different time-depth relationships and their impact on estimated depth to crater floor. The green trend constrained by a synthetic seismogram gives the most confident well-to-seismic tie.

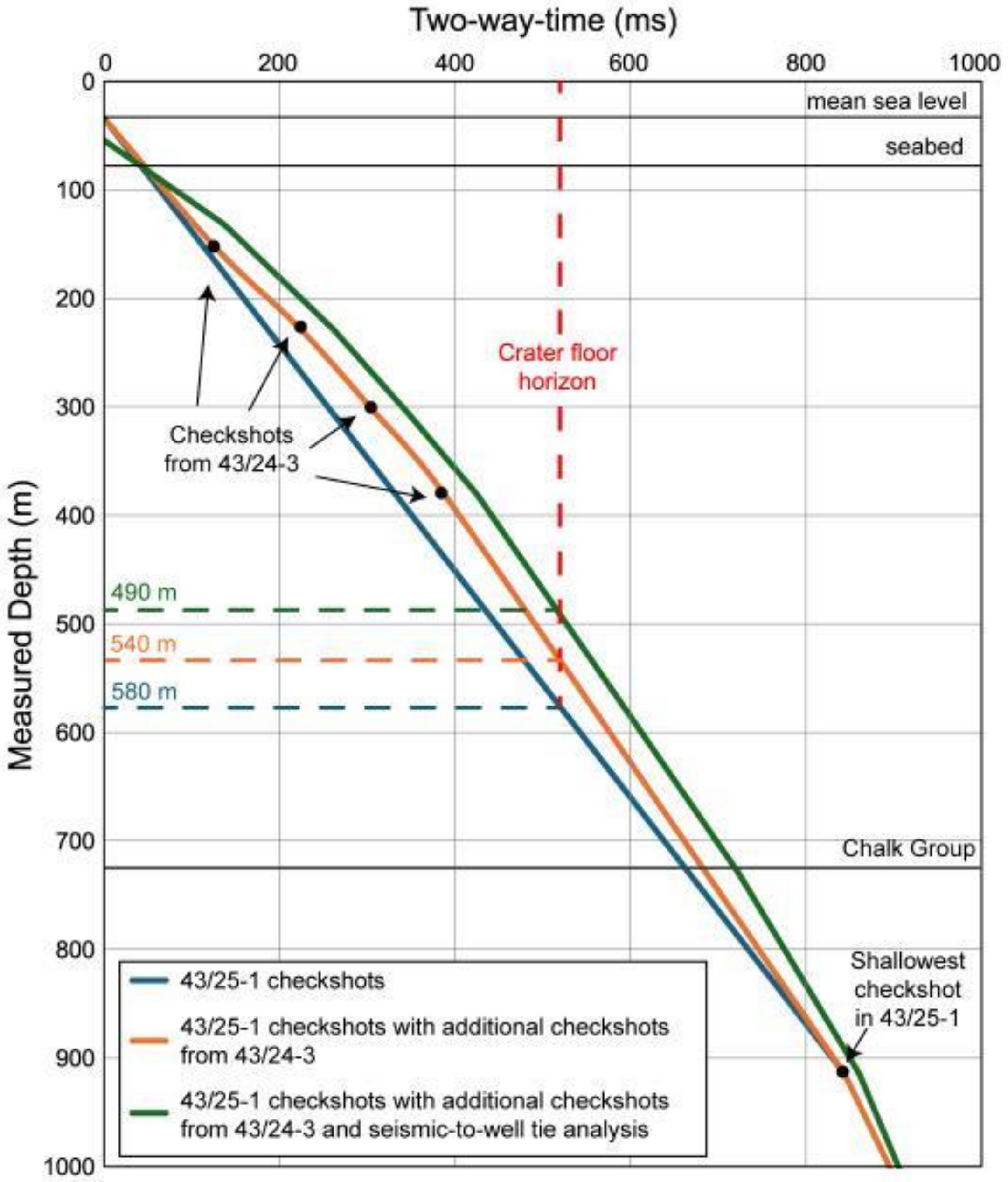

Figure S3. Full well-to-seismic tie for the 43/25-1 well.

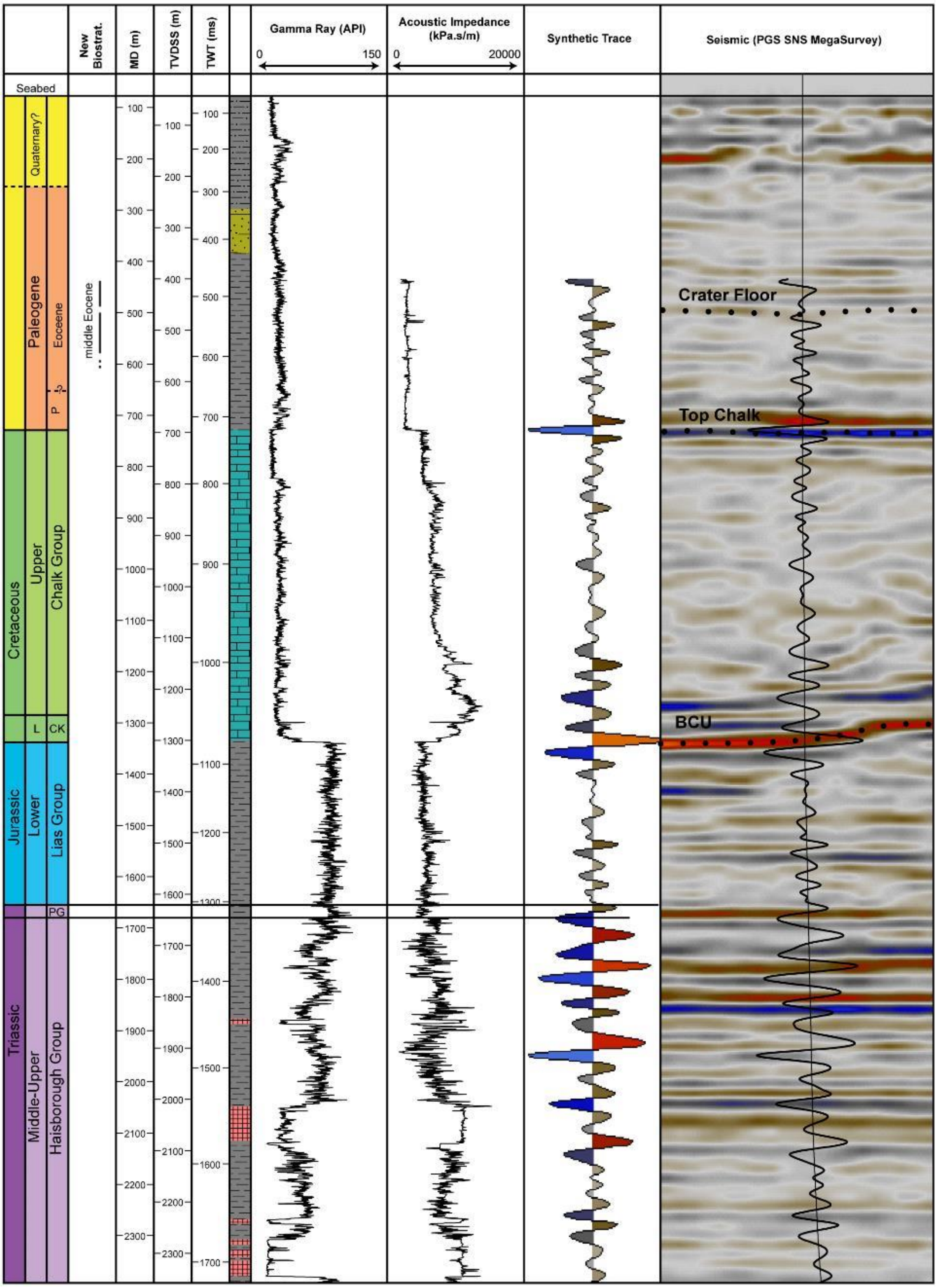

Figure S4. Photomicrographs of smear slides from samples at 500 m MD - characteristic of background middle Eocene samples - and 494 m MD: a-b) XPL images of 500 m showing very good nannofossil preservation with little background abiogenic carbonate; c) PPL image of 500 m showing clay particles, opaque minerals, organic material and diffuse micron-scale (opaque) pyrite; d-e) XPL image of 494 m showing abundant abiogenic, high birefringence carbonate grains; f) PPL image of 494 m showing relative scarcity of organic material, clays and pyrite. Bottom two rows: XPL images of example nannofossil specimens observed in other 3 other cuttings samples from a depth of 494 m; these specimens are also characteristic of the preservation and diversity through other middle Eocene samples studied.

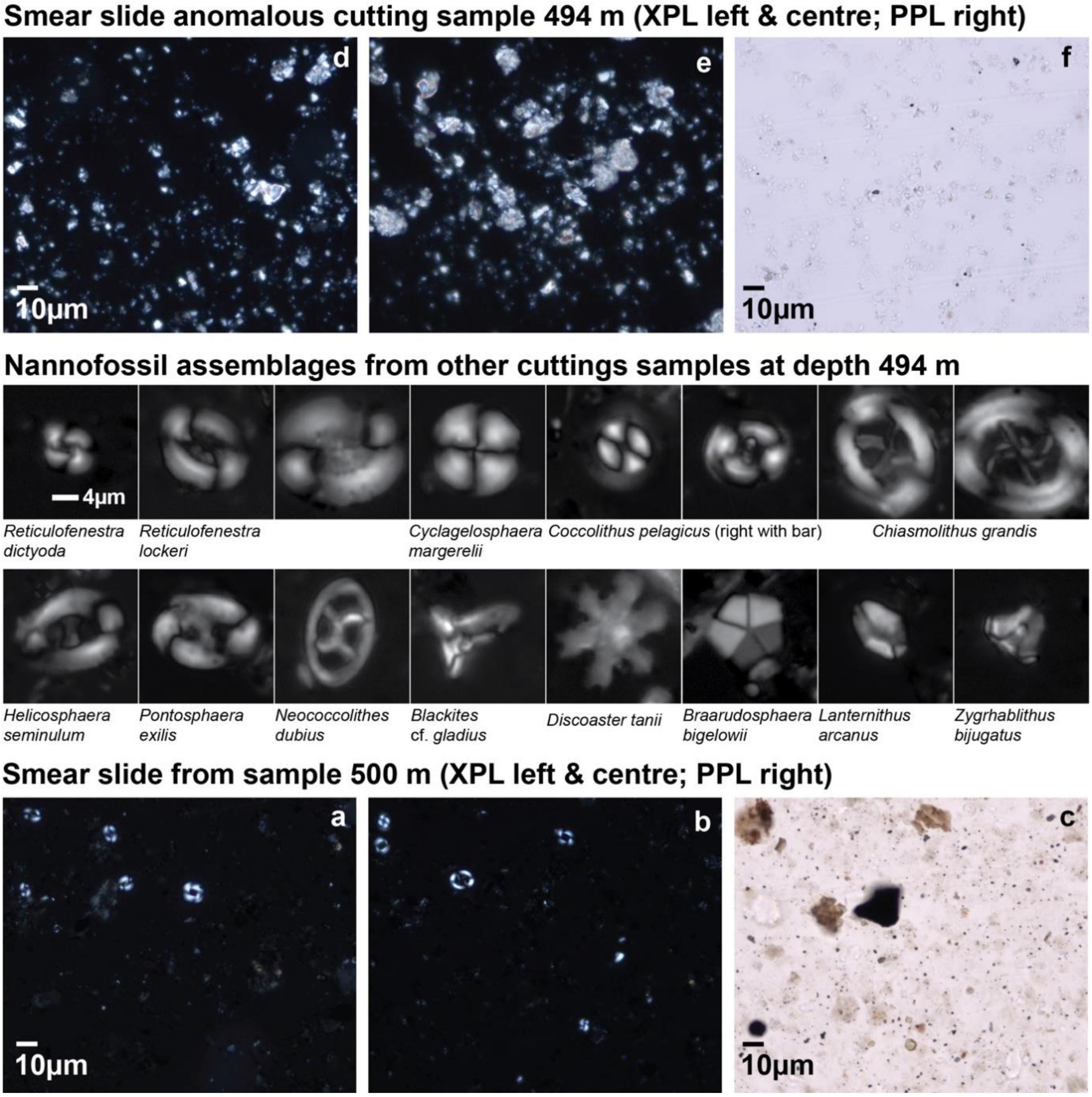

Supplementary text

Paleogene microfossil assemblages from 43/25-1 well: The assemblages at 183 m consists mostly of reworked Cretaceous nannofossils. Samples between 219-433 m consist of rare to very rare Cenozoic nannofossils, mostly non-age diagnostic small reticulofenestrids. The assemblage is dominated by reticulofenestrid coccoliths, but includes *Sphenolithus furcatolithoides* and not *Reticulofenestra umbilicus,* which constrains the age range of these sediments to be between 45.95 (base *S. furcatolithoides*) and 43.06 Ma (top *R. umbilicus*), equivalent to Zones CNE10-12 (Agnini et al. 2024) or NP15-lower NP16 (Martini et al. 1971) The base of *S. furcatolithoides* (45.95 Ma) is placed between depths 597 and 603 m. The two samples between 603-610 m depth contain nannofossil assemblages with similar abundance and diversity to the interval above, but without *S. furcatolithoides.* Without distinctive markers they are hard to date precisely, although most likely are still middle Eocene in age. The one sample barren of nannofossils between 439 and 610 m is at depth 494 m, with a smear slide dominated by high-birefringence particles of ~2 to 15 µm diameter, in cross-polarized light (XPL) (Fig. S4), interpreted to be carbonate. In plain polarized light (PPL) this smear slide differs from the surrounding middle Eocene sediments in having little organic matter or clay particles, and substantially less micron-scale pyrite, which is pervasive in the background of other middle Eocene samples (Fig. S4). In XPL, silt-grade multi-domain, low birefringence particles were commonly observed that were not present in other samples (Figure S4).

Petrography of cuttings from 43/25-1 well: Rocks are predominantly shales with varying amount of quartz, with transitions to quartz wackes (524-500 m) and mudstones (421-549 m). In the profile, there is a trend of finer grain sizes with depth. The shales are poorly sorted and are dominated by clay to coarse silt grainsize, but coarser quartz and feldspar grains are present as well. Grains are mostly angular. The amount of fine-grained clayey matrix increases with depth but mudstone intervals also exist at higher levels. The presence of iron bearing phases such as limonite and goethite cause a brownish color to the rocks. A shape preferred orientation of grains and phyllosilicates occurs at several depth intervals (451, 561 m). At a depth of 494 m, abundant sub-rounded and elongated, black carbonate pebble aggregates are evident. A turbulent flow regime with eddies is demonstrated at 500 m by the alignment of micas. Below 506 m cuttings show more homogeneous sedimentation without strong variations in lithologies. The main minerals in the rocks are quartz, feldspar, mica, glauconite, clay phases, calcite, iron oxides. Gypsum plates were recorded locally.

Supplementary references:

Agnini, C., Fornaciari, E., Raffi, I., Catanzariti, R., Pälike, H., Backman, J., Rio, D., 2014. Biozonation and biochronology of Paleogene calcareous nannofossils from low and middle latitudes. Newsletters on Stratigraphy 47, 131–181.

Martini, E., 1971. Standard Tertiary and Quaternary calcareous nannoplankton zonation. In: Farinacci, A. (Ed.), Proceedings 2nd International Conference Planktonic Microfossils Roma: Rome (Ed. Tecnosci.) 2, 739–785.
