# Supplementary material for: Multiple lines of evidence for a hypervelocity impact origin for the Silverpit Crater
Source: Nat Commun. 2025 Sep 20;16:8312. doi: 10.1038/s41467-025-63985-z (PMC12450236; doi:10.1038/s41467-025-63985-z)
Supplement: Supplementary file 2 — Description of Additional Supplementary File [file 41467_2025_63985_MOESM2_ESM.pdf]

Description of Additional Supplementary File

Movie S1: Numerical simulation of Silverpit impact event.
